# Accelerometer-measured moderate-to-vigorous physical activity is associated with reduced risk of bowel resection and mortality in inflammatory bowel disease: a prospective cohort study

**DOI:** 10.1097/JS9.0000000000003255

**Published:** 2025-11-04

**Authors:** Zixuan He, Yuhao Sun, Hanyi Huang, Judith Wellens, Yilong Liu, Lintao Dan, Xixian Ruan, Tian Fu, Zhaoshen Li, Xiaoyan Wang, Xue Li, Susanna C. Larsson, Johan Burisch, Jie Chen, Yu Bai, Fernando Magro

**Affiliations:** a Department of Gastroenterology, Changhai Hospital, Naval Medical University, Shanghai, China; b Changhai Clinical Research Unit, Changhai Hospital, Naval Medical University, Shanghai, China; c National Key Laboratory of Immunity and Inflammation, Naval Medical University, Shanghai, China; d Department of Gastroenterology, The Third Xiangya Hospital, Central South University, Changsha, China; e Department of Gastroenterology, The Second Affiliated Hospital of Zhejiang University School of Medicine, Hangzhou, China; f Leuven University Hospital, Department of Gastroenterology and Hepatology, Leuven, Belgium; g College of Basic Medicine Sciences, Naval Medical University, Shanghai, China; h Department of Gastroenterology, Ruijin Hospital, School of Medicine, Shanghai Jiao Tong University, Shanghai, China; i Department of Gastroenterology, Affiliated Hangzhou First People’s Hospital, Westlake University Medical College, Hangzhou, China; j Department of Big Data in Health Science School of Public Health and the Second Affiliated Hospital, Zhejiang University School of Medicine, Hangzhou, China; k Unit of Cardiovascular and Nutritional Epidemiology, Institute of Environmental Medicine, Karolinska Institutet, Stockholm, Sweden; l Unit of Medical Epidemiology, Department of Surgical Sciences, Uppsala University, Uppsala, Sweden; m Gastro Unit, Medical Division, Copenhagen University Hospital - Amager and Hvidovre, Hvidovre, Denmark; n Copenhagen Center for Inflammatory Bowel Disease in Children, Adolescents and Adults, Copenhagen University Hospital - Amager and Hvidovre, Hvidovre, Denmark; o Department of Clinical Medicine, Faculty of Health and Medical Sciences, University of Copenhagen, Copenhagen, Denmark; p Postdoctoral Station of Clinical Medicine, the Third Xiangya Hospital, Central South University, Changsha, China; q Xiangya School of Public Health, Central South University, Changsha, China; r CINTESIS@RISE, Department of Community Medicine, Information and Health Decision Sciences, Faculty of Medicine of the University of Porto, Porto, Portugal

**Keywords:** accelerometry, bowel resection, inflammatory bowel disease, moderate-to-vigorous physical activity, mortality, prognosis

## Abstract

**Background:**

There is limited consensus on physical activity recommendations for inflammatory bowel disease (IBD) patients due to insufficient high-quality evidence.

**Methods:**

We collected data from 1303 UK Biobank participants with IBD diagnosis and device-measured physical activity. Moderate-to-vigorous physical activity (MVPA) was classified based on data measured by wrist-worn accelerometers over a 7-day period. MVPA patterns were defined as inactive, active weekend warrior, and regularly active.

**Results:**

During a median follow-up of 7.8 years, 56 incident bowel resection cases and 86 deaths were documented. Compared to those in the lowest tertile, participants in the highest tertile of MVPA duration had lower risks of bowel resection [hazard ratio (HR), 0.44; 95% confidence interval (CI), 0.22–0.86] and mortality (HR, 0.49; 95% CI, 0.27–0.89). MVPA duration showed a linear association with bowel resection, while its dose–response relationship with mortality plateaued at approximately 58 minutes/day. The active weekend warrior pattern was inversely associated with bowel resection (HR, 0.28; 95% CI, 0.12–0.65), and the regularly active pattern was inversely associated with both bowel resection (HR, 0.37; 95% CI, 0.19–0.69) and mortality (HR, 0.53; 95% CI, 0.31–0.91) compared to the inactive group. The findings remained consistent after individually adjusting for C-reactive protein, Charlson Comorbidity Index, disease severity, baseline disease activity status, use of IBD-related medications, and baseline bowel resection history.

**Conclusion:**

Longer durations of accelerometer-measured MVPA were associated with reduced bowel resection risk and mortality. For affected individuals, the regularly active pattern may be the optimal choice, although the active weekend warrior pattern still provides health benefits compared to being inactive.

## Graphical abstract

**Figure:**
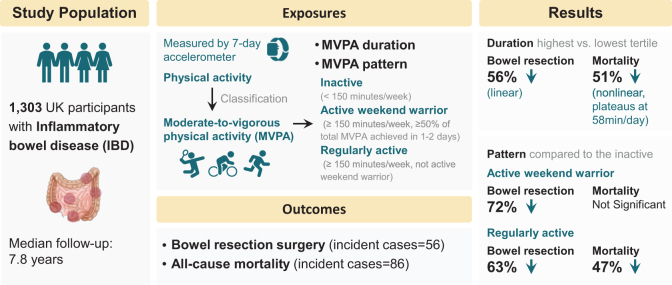

## Introduction

Inflammatory bowel disease (IBD), comprising Crohn’s disease (CD) and ulcerative colitis (UC), is a chronic inflammatory condition of the gastrointestinal tract influenced by both genetic and lifestyle factors[1]. It significantly impairs patients’ quality of life and is associated with considerable morbidity[2]. Managing IBD typically requires long-term medication, with many patients eventually requiring surgical intervention[3]. However, these medical treatments are not effective for all patients, and they impose a significant economic burden on both individuals and healthcare systems^[4,5]^. A previous study reported that 30–50% of patients with IBD seek complementary and alternative medicines including physical activity[6].

Current evidence indicates a link between exercise and IBD, suggesting that regular physical activity may benefit patients by releasing protective myokines, inducing an anti-inflammatory environment, improving gut microbiota, and reducing the risk of IBD-specific complications such as osteoporosis^[7,8]^. However, concerns exist regarding excessive and strenuous physical activity, which might exacerbate gastrointestinal symptoms[6]. Existing guidelines lack consensus on specific exercise recommendations regarding intensity, duration, pattern, as well as long-term benefit and safety. Only one guideline encourages clinicians to assess patients’ activity levels, identify and address barriers to physical activity, and promote increased activity within patients’ tolerance limits[9]. The paucity of studies on the role of physical activity among IBD patients poses challenges for healthcare providers and patients in determining optimal exercise regimens for managing the disease as a stand-alone intervention or as part of a comprehensive lifestyle modification program. To be specific, current studies primarily focus on short-term outcomes, offering limited insight into long-term prognostic outcomes such as surgery and mortality. Furthermore, no high-quality evidence exists confirming whether differences exist between exercise patterns. In addition, previous studies often had limited sample sizes and primarily relied on self-reported data to evaluate physical activity, which is susceptible to recall bias and may not accurately reflect actual activity levels ^[7,10–12]^.

To provide a more comprehensive and objective understanding of the role, safety and benefit of moderate-to-vigorous physical activity (MVPA) on IBD prognosis, this study investigated whether higher duration and specific patterns of objectively measured MVPA are associated with a reduced risk of bowel resection surgery and all-cause mortality in individuals with IBD. This cohort study has been reported in line with the STROCSS guidelines[13].

**HIGHLIGHTS**

- Longer objectively measured moderate-to-vigorous physical activity (MVPA) is linked to lower bowel resection risk and mortality in individuals with inflammatory bowel disease (IBD).
- The guideline of at least 150 minutes of MVPA per week, recommended for healthy individuals, may be also applicable to those with IBD.
- Compared to the inactive, the active weekend warrior pattern was inversely associated with bowel resection risk, while the regularly active pattern was inversely associated with both bowel resection risk and mortality.

## Methods

### Study population

Participants in this study were from the UK Biobank, a large prospective cohort designed to allow detailed investigation of risk factors for a wide range of diseases. It involved half a million participants aged 40–69 years at recruitment in 2006–2010^[14,15]^. Participants were initially assessed at 22 assessment centers across the UK via touchscreen questionnaires, face-to-face interviews, physical measurements, and biological samples. Additional assessments were conducted to enhance phenotyping, including web-based questionnaires and accelerometry. Embedded within the UK’s National Health Service (NHS), the UK Biobank tracks the long-term health of participants and identifies disease outcomes using routine medical records[16]. The study of the UK Biobank received ethical approval from the North West Multi-centre Research Ethics Committee (Ref 21/NW/0157). All participants provided written informed consent.

**Figure 1.**
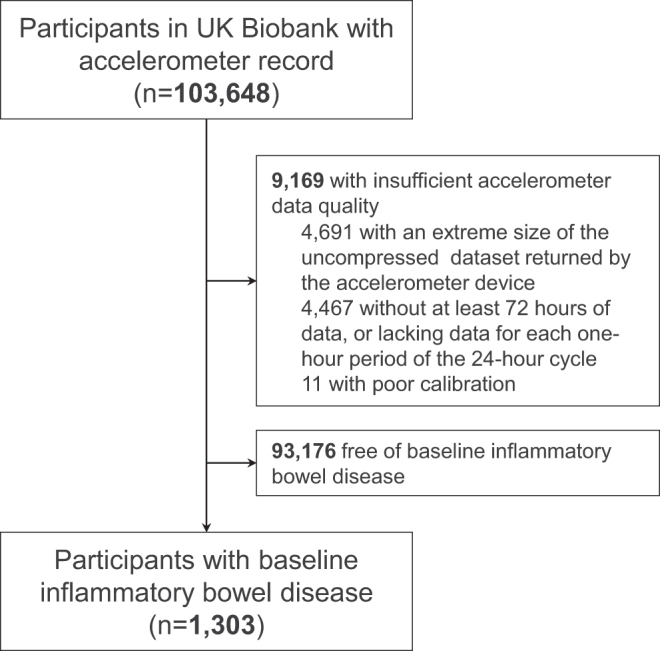
Flow chart of the study.

Our study initially recruited 103 648 participants with accelerometer data. After excluding 9169 participants with insufficient accelerometer data quality and 93 176 participants without baseline diagnosed or self-reported IBD, a total of 1303 individuals with both valid device-measured physical activity and baseline IBD were included (Fig. 1). This study followed the STROBE guidelines for cohort studies.

### Measurement of MVPA

Physical activity was assessed in approximately 100 000 UK Biobank participants during 2013–2015 using Axivity AX3 triaxial accelerometers worn on the dominant wrist for 7 consecutive days, and the study demonstrated excellent compliance, with over 93% providing more than 72 hours of wear time and no missing data bias observed by time of day^[17,18]^. Wrist accelerometry using Axivity AX3 has demonstrated validity in estimating activity energy expenditure (*r* = 0.64) and total energy expenditure (*r* = 0.90) when compared to the gold standard of doubly labelled water[19]. Additionally, within the UK Biobank study, baseline accelerometer measures showed moderate-to-high reproducibility, with intraclass correlation coefficients (ICCs) of 0.75 for overall activity and 0.60 for MVPA over 3.7 years[20].

The Axivity AX3 device has been validated in a previous study using multi-axis shaking tests and showed equivalent output to GENEActive accelerometer[21], which has been validated in both laboratory and free-living assessments^[22,23]^. Triaxial acceleration data are collected at 100 Hz with a dynamic range of ±8 g and extracted after processing[18]. Further, accelerometer data were classified into movement behaviors (sleep, sedentary behavior, light physical activity, and MVPA) using machine-learning methods that have been validated in the UK sample[24]. MVPA is defined as the physical activity performed at over 3 metabolic equivalents of task (METs), where 1 MET represents the energy expenditure of an individual at rest. Non-wear periods are imputed using time-matched averages from other days to account for potential diurnal bias in wear patterns and a physical activity outcome variable is finally constructed by averaging all worn and imputed values for each participant[18]. MVPA duration was categorized into tertiles: low, medium, and high. MVPA patterns were classified based on guideline-based threshold[25] into three categories: inactive (<150 minutes/week), active weekend warrior (≥150 minutes/week with ≥50% of MVPA achieved in any 1–2 days of the week), and regularly active (≥150 minutes/week but not active weekend warrior)^[26,27]^.

### Outcome assessment

Bowel resections were identified through operational records from hospital inpatient data of the NHS. All operations and procedures are recorded using OPCS-4 (Offices of Population, Censuses and Surveys: Classification of Interventions and Procedures version 4) codes. Corresponding OPCS-4 codes are provided in Supplemental Digital Content Table S1, available at: http://links.lww.com/JS9/F433^[28,29]^. Deaths were documented through linkage to the national death register. Follow-up began on the date when all accelerometer measurements were completed and continued until the first occurrence of bowel resection, death, loss to follow-up, or the last date of hospital admission (31 October 2022 for England; 31 August 2022 for Scotland; and 31 May 2022 for Wales).

### Assessment of covariates

Covariates were selected based on prior knowledge[28], including age in years, sex (male/female), ethnicity (White/others), education attainment (college and above/below college), Townsend deprivation index (TDI), employment (employed/unemployed), body mass index (BMI), smoking status (ever/never), drinking status (current/non-current), and adherence to a healthy diet (yes/no). TDI was derived from participants’ postcodes, with a higher score indicating greater deprivation. Adherence to a healthy diet was defined as meeting at least four out of seven criteria related to the consumption of fruits, vegetables, fish, unprocessed red meats, whole grains, refined grains, and processed meats.^[30,31]^

In sensitivity analyses, we additionally adjusted for C-reactive protein (CRP), self-reported overall health (good, fair, or poor), waist–hip ratio (WHR), Charlson Comorbidity Index[32], and several IBD-related factors, including disease severity (as a time-varying covariate)^[33,34]^, baseline disease activity status (active/inactive)[33], common IBD-related medications (use/not use)[28], baseline bowel resection history (yes/no), and surgical event (no surgical event, elective surgery, emergency surgery)[35]. Disease severity was defined by whether participants experienced IBD-related hospitalizations or surgeries, with severe periods considered as the 3 months before and after each event. Baseline disease activity status indicates whether any IBD-related hospitalization or surgery occurred within 1 year before or after baseline. Detailed information on covariates is presented in Supplemental Digital Content Table S2, available at: http://links.lww.com/JS9/F433.

### Statistical analysis

Baseline characteristics by tertile of MVPA duration are presented as means ± standard deviations (SDs) for continuous variables and counts (percentages) for categorical variables. Missing values (<0.84% for all covariates) were imputed using the median for continuous variables and mode for categorical variables (Supplemental Digital Content Table S2, available at: http://links.lww.com/JS9/F433). Associations between MVPA and IBD prognosis were estimated using two multivariable Cox regression models. Model 1 was adjusted for age and sex. Model 2 was further adjusted for ethnicity, education, TDI, employment, BMI, smoking status, drinking status, and adherence to a healthy diet. The proportional hazards assumption was tested based on Schoenfeld residuals (all models satisfied the assumption with *P* > 0.15). We used *E*-value to assess the potential impact of unmeasured confounding on observed associations from main models[36]. The potential non-linear association between MVPA duration and IBD prognosis was tested using restricted cubic spline with knots at the 10th, 50th, and 90th percentiles of MVPA duration, using 0 as the reference[37]. Nonlinearity in exposure–outcome relationships was evaluated by likelihood ratio tests.

To correct for regression dilution bias, a systematic underestimation of epidemiological associations caused by measurement error and temporal variability in MVPA level, we adjusted the effect estimates using the previously reported ICC of 0.60[20]. Subgroup analyses assessed interactions by age (>60 years/≤ 60 years), sex (male/female), disease duration (>15 years/≤ 15 years), BMI (>30/≤ 30), smoking status (non-smoker/ever-smoked), and adherence to a healthy diet (yes/no), with multiplicative and additive interactions evaluated through interaction terms and relative excess risk due to interaction, respectively[38]. Additionally, we explored the associations of MVPA with the prognosis of CD and UC separately.

Several sensitivity analyses were performed to further test the robustness of the results: (1) we employed multiple imputation to address missing values; (2) participants who had the outcome of interest within 1 year and within 2 years of follow-up were excluded to minimize reverse causation; (3) separate analyses were performed with additional adjustments made for CRP, self-reported health, WHR, Charlson Comorbidity Index, use of IBD-related medications, disease severity, baseline disease activity status, baseline bowel resection history, and surgical event, respectively. All analyses were performed using R software, version 4.2.1. A two-tailed *P* < 0.05 was considered statistically significant.

## Results

**Figure 2.**
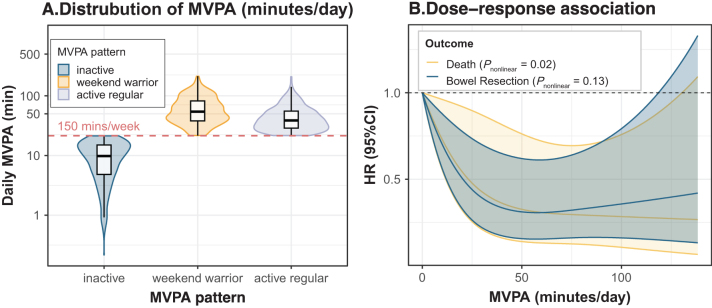
Distribution of daily MVPA (A) and dose–response associations of daily MVPA with mortality and bowel resection risk (B). MVPA, moderate-to-vigorous physical activity; HR, hazard ratio.

During a median follow-up of 7.8 years, we recorded 56 bowel resections and 86 all-cause deaths of the 1303 participants included. Baseline characteristics stratified by MVPA duration and pattern are shown in Table 1 and Supplemental Digital Content Table S3, available at: http://links.lww.com/JS9/F433, respectively. 506 (38.8%) participants were classified as inactive, 287 (22.0%) as active weekend warriors, and 510 (39.1%) as regularly active. Individuals who undertook more MVPA were generally younger, more likely to be male, employed, had higher educational attainment, lower BMI, lower inflammation level, and better self-reported health. No notable differences in IBD subtypes or disease-specific characteristics were observed across MVPA durations or patterns. Figure 2A shows the distribution of MVPA duration across different MVPA patterns. The median MVPA time was 64.6 minutes/week for the inactive group, 380.8 minutes/week for active weekend warriors, and 271.3 minutes/week for regular exercisers. Participants exhibited no significant difference in MVPA duration across baseline disease activity statuses (*P* = 0.28) (Supplemental Digital Content Figure S1, available at: http://links.lww.com/JS9/F433). The mean MVPA showed a negligible trend (β = 9.6 × 10^−5^ minutes/day, *P* < 0.001) over the 7-day monitoring period (Supplemental Digital Content Figure S3, available at: http://links.lww.com/JS9/F433).

**Table 1:** Baseline characteristics of participants stratified by tertile of moderate-to-vigorous physical activity duration

Characteristics	Overall (*n* = 1303)	MVPA duration, minutes/day		
		Tertile 1 (>0 to ≤17) (*n* = 435)	Tertile 2 (>17 to ≤40.7) (*n* = 435)	Tertile 3 (>40.7) (*n* = 433)
Age, year	56.6 ± 7.7	57.3 ± 7.7	56.5 ± 8.0	55.9 ± 7.4
Female (%)	701 (53.8)	277 (63.7)	246 (56.6)	178 (41.1)
White (%)	1267 (97.3)	424 (97.7)	419 (96.3)	424 (97.9)
College and above (%)	478 (37.0)	128 (29.6)	152 (35.3)	198 (46.0)
Townsend deprivation index	−1.60 ± 2.88	−1.61 ± 2.92	−1.51 ± 2.97	−1.69 ± 2.74
Employed (%)	799 (61.5)	246 (56.8)	269 (61.8)	284 (65.7)
Body mass index, kg/m^2^	26.81 ± 4.66	28.44 ± 5.49	26.55 ± 4.24	25.45 ± 3.54
Ever smoking (%)	655 (50.3)	247 (56.9)	205 (47.1)	203 (46.9)
Current drinking (%)	1215 (93.3)	390 (89.9)	408 (93.8)	417 (96.3)
Adherence to a healthy diet (%)	847 (65.3)	256 (59.1)	292 (67.4)	299 (69.4)
C-reactive protein, mg/L	3.41 ± 5.72	4.42 ± 6.78	3.15 ± 5.08	2.68 ± 4.98
Self-reported health (%)				
Good	794 (61.2)	219 (50.7)	278 (64.2)	297 (68.8)
Fair	383 (29.5)	140 (32.4)	133 (30.7)	110 (25.5)
Poor	120 (9.3)	73 (16.9)	22 (5.1)	25 (5.8)
Waist-to-hip ratio	0.86 ± 0.09	0.87 ± 0.09	0.86 ± 0.08	0.87 ± 0.08
**IBD subtype (Montreal Classification)**				
Crohn’s disease				
L1 (small bowel disease or terminal ileitis)	68 (5.2)	19 (4.4)	25 (5.7)	24 (5.5)
L2 (colon)	105 (8.1)	34 (7.8)	39 (9.0)	32 (7.4)
L3/LX (ileocecal Crohn’s disease or location not defined)	251 (19.3)	94 (21.6)	83 (19.1)	74 (17.1)
Ulcerative colitis				
E1 (ulcerative proctitis)	105 (8.1)	27 (6.2)	36 (8.3)	42 (9.7)
E2 (left-sided UC)	73 (5.6)	22 (5.1)	20 (4.6)	31 (7.2)
E3 (extensive UC)	45 (3.5)	11 (2.5)	13 (3.0)	21 (4.8)
EX (extent not defined)	656 (50.3)	228 (52.4)	219 (50.3)	209 (48.3)
**IBD-specific characteristics**				
Disease duration, years	20.3 ± 14.1	20.6 ± 14.3	19.9 ± 13.9	20.5 ± 14.1
Active baseline disease activity status (%)	353 (27.1)	126 (29.0)	116 (26.7)	111 (25.6)
Common IBD-related medications use (%)	484 (37.1)	173 (39.8)	150 (34.5)	161 (37.2)

IBD, inflammatory bowel disease; MVPA, moderate-to-vigorous physical activity; UC, ulcerative colitis.

As shown in Table 2, after adjusting for a wide range of sociodemographic and lifestyle factors, we found that higher MVPA duration was associated with reduced bowel resection risk [hazard ratio (HR) per 1 SD, 0.67; 95% confidence interval (CI), 0.47–0.96] and mortality (HR per 1 SD, 0.71; 95% CI, 0.53–0.96). Compared to those in the lowest tertile, participants in the highest tertile of MVPA duration had an HR of 0.44 for bowel resection risk (95% CI, 0.22–0.86), and an HR of 0.49 for mortality (95% CI, 0.27–0.89). Restricted cubic spline analyses revealed no non-linear association between MVPA duration and bowel resection risk (*P*_non-linear_ = 0.13, Fig. 1B). However, a non-linear, reverse J-shaped relationship was observed between MVPA duration and mortality, with a potential plateau in risk reduction beyond approximately 58 minutes/day (*P*_non-linear_ = 0.02, Fig. 1B). Regarding MVPA patterns, the weekend warrior pattern was associated with a reduced risk of bowel resection (HR, 0.28; 95% CI, 0.12–0.65) but not with mortality (HR, 0.79; 95% CI, 0.44–1.42) compared to the inactive. The regularly active pattern was inversely associated with the risks of bowel resection (HR, 0.37; 95% CI, 0.19–0.69) and mortality (HR, 0.53; 95% CI, 0.31–0.91) (Table 2).

**Table 2:** Associations of moderate-to-vigorous physical activity with bowel resection risk and mortality

	Cases/person-years^a^	Model 1^b^		Model 2^c^		E-value
		HR (95% CI)	*P*	HR (95% CI)	*P*	
**MVPA duration, min/day**						
*Bowel resection risk*						
Per 1-SD increment		**0.71** **(0.51, 0.99)**	**0.043**	**0.67 (0.47, 0.96)**	**0.028**	2.35 (1.25, 3.68)
Tertile 1	28/3176	1 (reference)		1 (reference)		
Tertile 2	12/3312	**0.39 (0.20, 0.78)**	**0.007**	**0.37 (0.18, 0.74)**	**0.005**	4.85 (2.04, 10.59)
Tertile 3	16/3331	**0.50 (0.27, 0.94)**	**0.031**	**0.44 (0.22, 0.86)**	**0.016**	3.97 (1.60, 8.56)
*P* for trend^d^			**0.021**		**0.012**	
*Mortality*						
Per 1-SD increment		**0.63 (0.47, 0.85)**	**0.002**	**0.71 (0.53, 0.96)**	**0.026**	2.17 (1.25, 3.18)
Tertile 1	42/3301	1 (reference)		1 (reference)		
Tertile 2	26/3366	**0.59 (0.36, 0.96)**	**0.035**	0.66 (0.40, 1.10)	0.114	-
Tertile 3	18/3395	**0.39 (0.22, 0.68)**	**0.001**	**0.49 (0.27, 0.89)**	**0.018**	3.50 (1.50, 6.67)
*P* for trend^d^			**0.001**		**0.015**	
**MVPA pattern**						
*Bowel resection risk*						
Inactive	33/3694	1 (reference)		1 (reference)		
Active weekend warrior	7/2200	**0.31 (0.14, 0.71)**	**0.006**	**0.28 (0.12, 0.65)**	**0.003**	6.60 (2.45, 16.15)
Regularly active	16/3924	**0.43 (0.23, 0.78)**	**0.006**	**0.37 (0.19, 0.69)**	**0.002**	4.85 (2.26, 10.00)
*Mortality*						
Inactive	46/3846	1 (reference)		1 (reference)		
Active weekend warrior	17/2232	0.64 (0.37, 1.14)	0.128	0.79 (0.44, 1.42)	0.434	-
Regularly active	23/3983	**0.45 (0.27, 0.74)**	**0.002**	**0.53 (0.31, 0.91)**	**0.020**	3.18 (1.43, 5.91)

MVPA, moderate-to-vigorous physical activity; HR, hazard ratio; CI, confidence interval; SD, standard deviation.

^a^ Person-years were calculated from the date when all accelerometer measurements were completed to the end of follow-up.

^b^ Model 1 adjusted for age and sex;

^c^ Model 2 adjusted for age, sex, education, Townsend deprivation index, employment, body mass index, smoking status, drinking status, and adherence to a healthy diet.

^d^ *P* for trend was calculated using the median of each tertile as a continuous variable.

^e^ Bold values highlight significant results.

**Figure 3.**
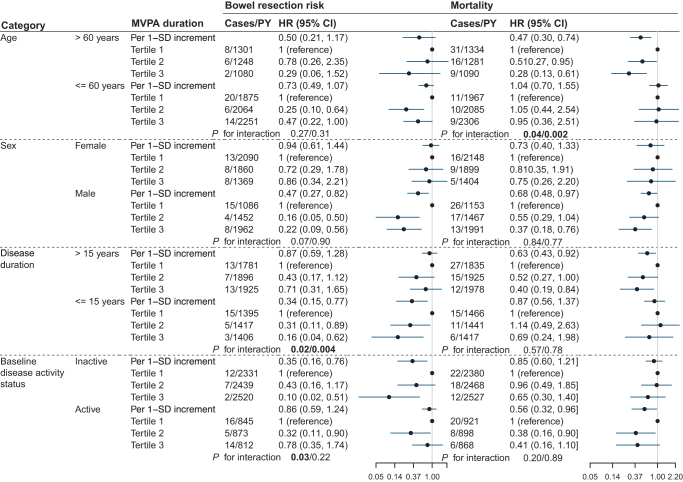
Associations of MVPA duration with bowel resection risk and mortality stratified by age, sex, disease duration, and baseline disease activity status. Model adjusted for age, sex, education, Townsend deprivation index, employment, body mass index, smoking status, drinking status, and adherence to a healthy diet, with exclusion of variables used for stratification. Person-years were calculated from the date when all accelerometer measurements were completed to the end of follow-up. The *P*-value for the interaction was presented as multiplicative/additive interaction. MVPA, moderate-to-vigorous physical activity; HR, hazard ratio; CI, confidence interval; SD, standard deviation; PY, person-years.

The protective benefit of MVPA on bowel resection risk was similar among individuals with CD and UC, while its benefit on mortality was more pronounced in those with UC (Supplemental Digital Content Table S5, available at: http://links.lww.com/JS9/F433). Analyses stratified by age, sex, disease duration, and baseline disease activity status are presented in Figure 3 and Supplemental Digital Content Figure S2, available at: http://links.lww.com/JS9/F433. In age-stratified analyses, the inverse associations between MVPA duration and mortality were more pronounced in individuals over 60 compared to those under 60 (*P*_interaction_ ≤ 0.04). There were no substantial differences by sex. When stratified by disease duration, the inverse associations between MVPA duration and bowel resection risk were more pronounced in those with IBD for less than 15 years (*P*_interaction_ ≤ 0.02). Additionally, MVPA duration was beneficial to individuals with both active and inactive baseline disease activity status but appeared particularly beneficial in reducing bowel resection risk among those with inactive baseline disease activity status (*P*_additive interaction_ = 0.03). We observed no significant interaction in the analyses stratified by BMI, smoking status, and adherence to a healthy diet (Supplemental Digital Content Table S6, available at: http://links.lww.com/JS9/F433).

Results were consistent, though a little attenuated, when we repeated analyses employing multiple imputation to address missing covariates, excluding participants who had the outcome of interest within 1 year and 2 years of follow-up, or in separate analyses additionally adjusted for CRP, self-reported health, WHR, Charlson Comorbidity Index, common IBD-related medications, disease severity, baseline disease activity status, prior history of bowel resection surgery, and surgical events (Supplemental Digital Content Tables S8 and S9, available at: http://links.lww.com/JS9/F433).[38]

## Discussion

### Interpretation

In this prospective cohort study, we comprehensively investigated the effects of both duration and pattern of accelerometer-measured MVPA on IBD prognosis. Our results indicate that longer durations of MVPA are linearly associated with reduced risks of bowel resection surgery, with per SD increment in duration corresponding to a 33% reduction in bowel resection risk. By contrast, the dose–response relationship between MVPA duration and mortality was nonlinear and appeared to reach a plateau at approximately 58 minutes/day. Compared with the inactive group, both the active weekend warrior pattern and regularly active pattern were associated with lower bowel resection risk, whereas only the regularly active pattern was associated with reduced mortality.

Understanding the influence of physical activity on long-term outcomes in IBD is of critical importance. However, high-quality studies are limited. Existing literature on physical activity and IBD prognosis, primarily based on self-reported data, supports the benefits of physical activity. Some small observational studies and RCTs have reported the potential short-term benefits of physical activity on individuals with IBD, including reduced disease activity^[10,39,40]^, improved quality of life[41], and improved psychological issues[42]. However, few studies have investigated the role of higher intensities of physical activity. A study on 242 IBD patients found that both self-reported walking and MVPA were independently associated with health-related quality of life, particularly higher volume of MVPA above 150 minutes/week and walking above 60 minutes/week[41]. The result supports the impact of MVPA on individuals with IBD. In addition, there is a lack of evidence on long-term prognostic outcomes. Only one cohort study focused on healthy lifestyle involving 363 CD patients and 465 UC patients found that higher self-reported physical activity was linked to lower all-cause mortality (HR for quintile 5 vs. quintile 1, 0.35; 95% CI, 0.19–0.65), which is in agreement with our results[43].

Despite similar overall MVPA volumes, only the regularly active pattern is linked to reduced mortality. The differential effects of the two activity patterns may stem from different driving factors of the outcome. Surgical risk is primarily associated with direct bowel injury and disease activity, whereas mortality is more closely linked to cardiovascular health, systemic inflammation, and comorbidities. Regular, evenly distributed activity better sustains lower systemic inflammation than concentrated activity, thereby offering better long-term mortality benefits. Notably, the regularly active group showed identical emergency surgery rates to the overall cohort (25% vs 25%). Subsequent analysis of mortality by surgical event revealed no significant differences (all *P* > 0.05; Supplemental Digital Content Figure S4, available at: http://links.lww.com/JS9/F433), suggesting that the reduced mortality rate is independently of surgical procedures. The present study suggests that a more evenly spread pattern of physical activity is the optimal choice for individuals with IBD. However, less frequent activity sessions, which may be easier to practice, still provide health benefits compared to being inactive.

### Strengths and limitations

The strengths of this study include its large sample size, extended follow-up period, excellent participant compliance, and the objective and unprecedented scale of accelerometry-based physical activity measurement. The study also benefited from comprehensive adjustment for important covariates and robust findings across multiple sensitivity analyses. By individually accounting for disease-related factors such as CRP levels, use of common IBD-related medications, disease severity, baseline disease activity status, and baseline bowel resection history, we aimed to minimize potential confounding effects of disease characteristics on the outcomes.

However, there are some limitations. First, physical activity was assessed only once over a 7-day period at baseline, which may not fully capture habitual activity patterns. However, prior research has demonstrated validated Axivity AX3 against doubly labelled water[19], and a single 7-day accelerometer assessment of physical activity provides a reproducible and practical measure over time. A recent study with 3138 UK Biobank participants in the main accelerometry substudy, who repeated the assessments up to four times quarterly after 3–4 years, reported ICCs of 0.75 for overall activity and 0.60 for MVPA[20]. Second, the study population was predominantly White (97.3%) with an average BMI of 26.8 ± 4.7 kg/m^2^, which may limit the generalizability of the findings to other racial/ethnic groups and underweight individuals. Future studies with more diverse demographic representation are warranted. However, the large size and diverse exposure metrics of the UK Biobank may still support a valid assessment of exposure–disease relationships[44]. Third, due to its observational nature, the study cannot establish causality. While we adjusted for multiple confounders, residual confounding by unmeasured factors cannot be completely ruled out. However, we calculated *E*-values to quantify the potential impact of unmeasured confounding. All between-group *E*-values exceeded 3.0, which exceed the measured effects of most known lifestyle confounders, supporting the robustness of our findings despite potential residual confounding (Table 2)[36]. Nevertheless, future RCTs are needed to confirm a causal relationship between MVPA and IBD prognosis.

### Clinical interpretations

To date, national organizations supporting individuals with IBD advocate for low-to-moderate exercise, including walking, treadmill running, bicycling, and swimming[45]. However, our findings challenge the current consensus for individuals with IBD in which they were only encouraged to safely participate in low-to-moderate exercise. According to our findings, the standard guidelines for healthy individuals, recommending at least 150 minutes of MVPA per week[25], may be equally applicable to individuals with IBD. Furthermore, the protective benefit of MVPA observed in individuals with longer disease duration and active baseline disease activity status suggests that MVPA may help prevent adverse outcomes even in those with advanced or active disease status. However, given the generally lower activity levels among IBD patients and potential variation by disease activity[46], the point estimate of our observed association may hold clinical relevance but should be interpreted with caution. Its applicability requires future large-scale validation and interventional data, particularly among patients in the active disease phase.

## Conclusions

Device-measured MVPA duration was associated with lower risks of bowel resection surgery and mortality. An active weekend warrior pattern, characterized by 1 or 2 sessions per week, is inversely associated with bowel resection risk, while a regularly active pattern is inversely associated with both bowel resection risk and mortality. The findings suggest that regular participation in MVPA may be a safe and effective strategy to improve long-term outcomes and mitigate adverse prognoses in individuals with IBD. Recommendations to increase MVPA as part of IBD management should be explored.

## Data Availability

The datasets analyzed during the current study are available in a public, open-access repository (https://www.ukbiobank.ac.uk/).

## Statistical expertise statement

The statistical analyses in this study were conducted under the guidance of epidemiologists Xue Li and Jie Chen.
